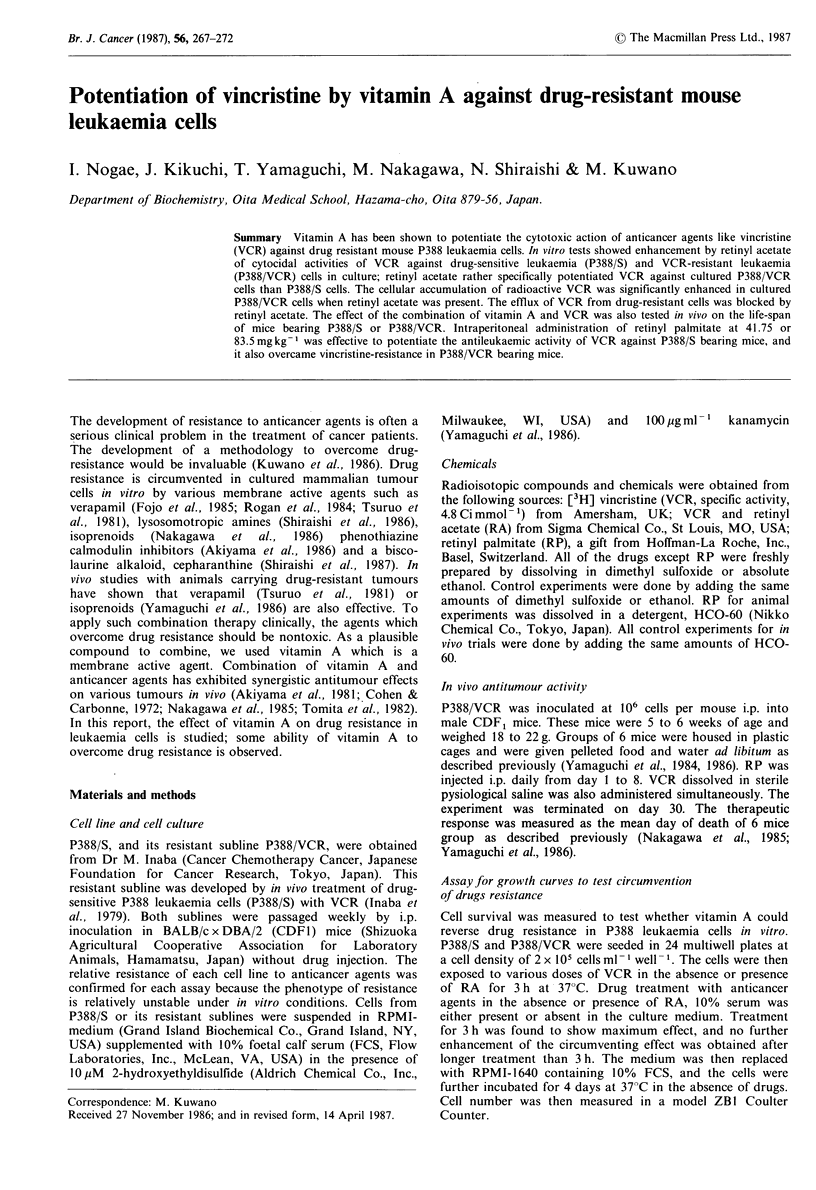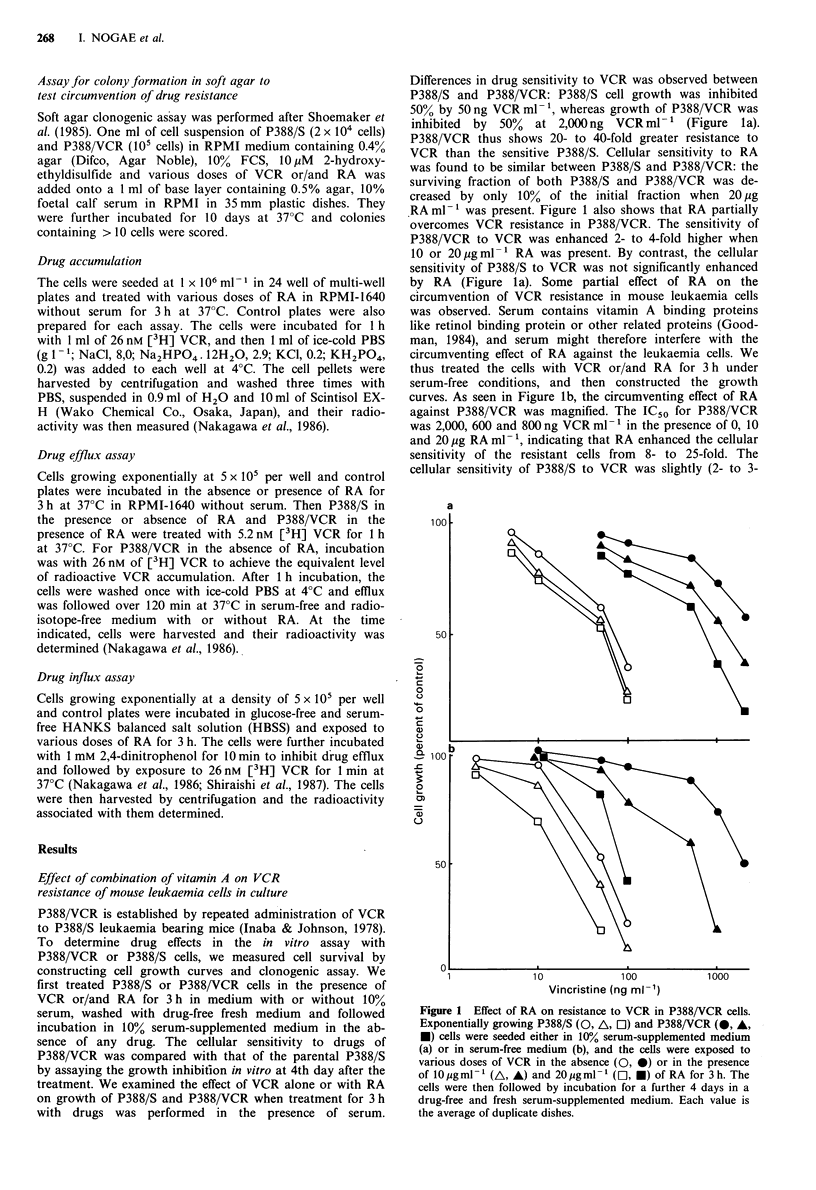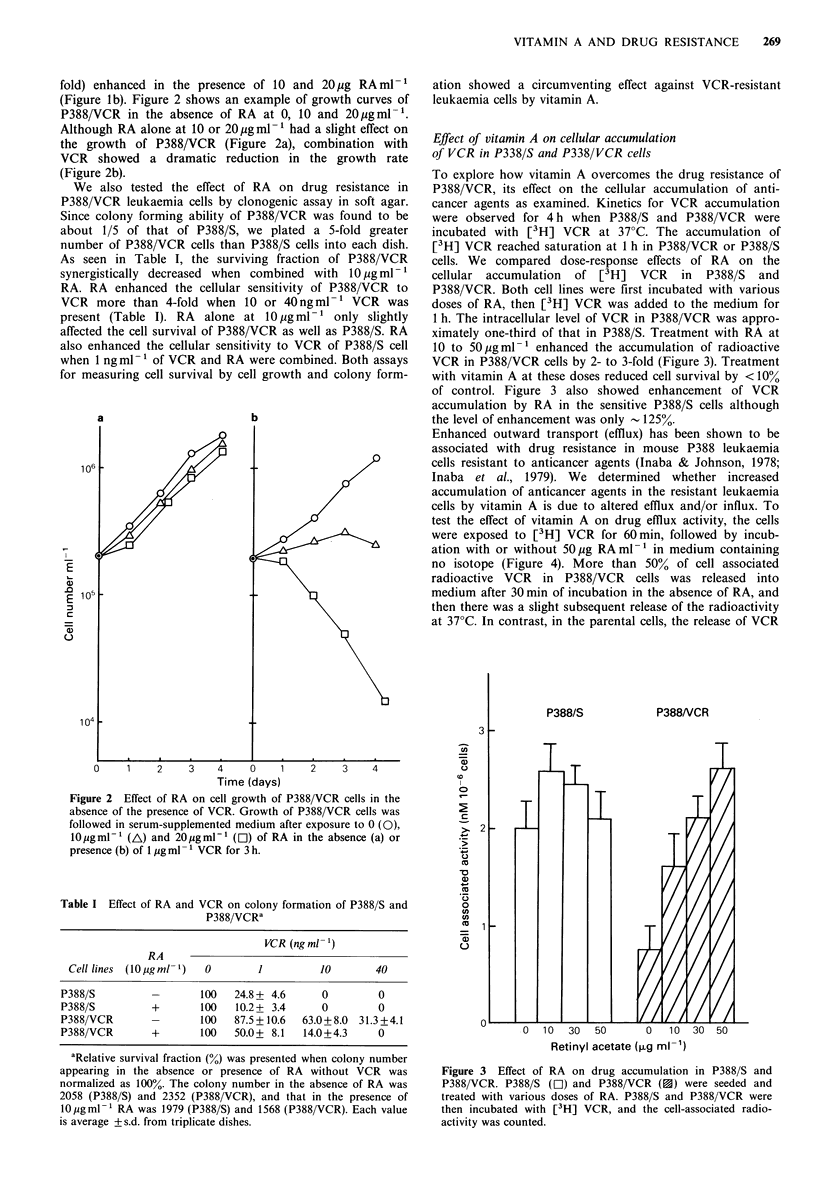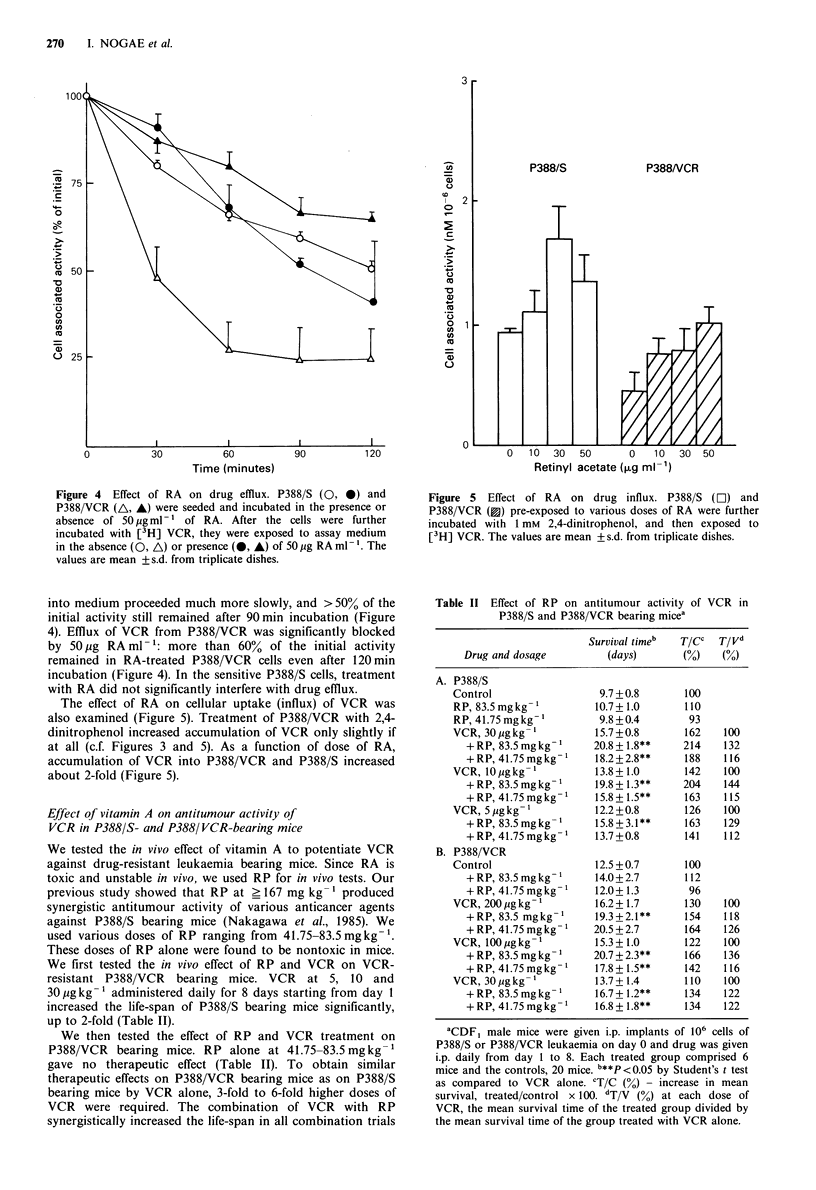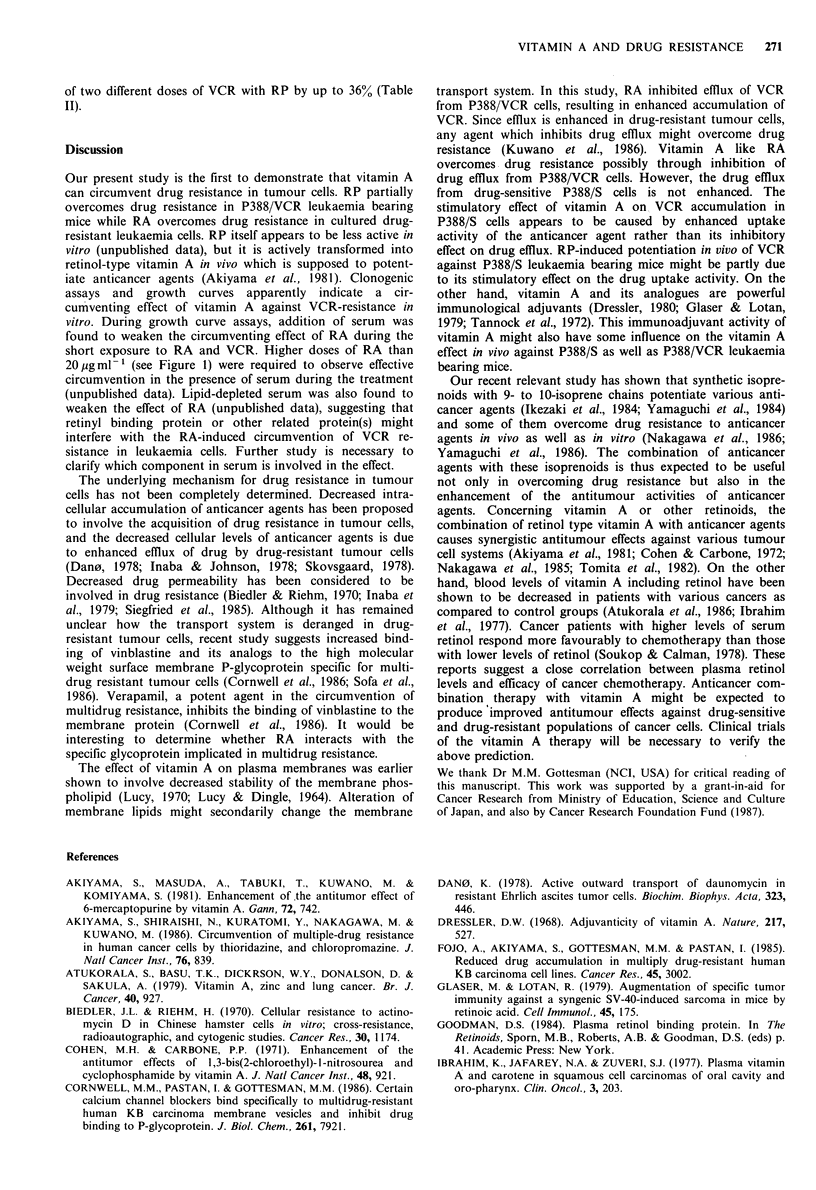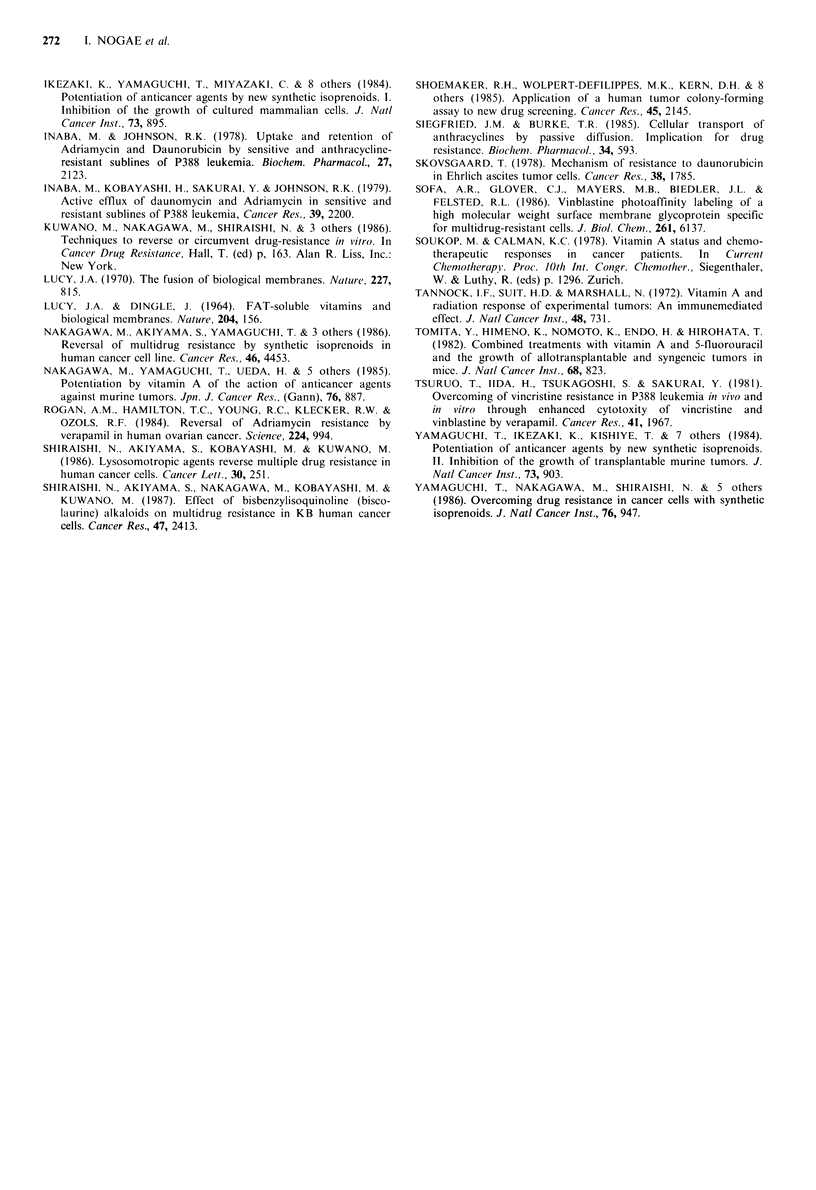# Potentiation of vincristine by vitamin A against drug-resistant mouse leukaemia cells.

**DOI:** 10.1038/bjc.1987.188

**Published:** 1987-09

**Authors:** I. Nogae, J. Kikuchi, T. Yamaguchi, M. Nakagawa, N. Shiraishi, M. Kuwano

**Affiliations:** Department of Biochemistry, Oita Medical School, Japan.

## Abstract

Vitamin A has been shown to potentiate the cytotoxic action of anticancer agents like vincristine (VCR) against drug resistant mouse P388 leukaemia cells. In vitro tests showed enhancement by retinyl acetate of cytocidal activities of VCR against drug-sensitive leukaemia (P388/S) and VCR-resistant leukaemia (P388/VCR) cells in culture; retinyl acetate rather specifically potentiated VCR against cultured P388/VCR cells than P388/S cells. The cellular accumulation of radioactive VCR was significantly enhanced in cultured P388/VCR cells when retinyl acetate was present. The efflux of VCR from drug-resistant cells was blocked by retinyl acetate. The effect of the combination of vitamin A and VCR was also tested in vivo on the life-span of mice bearing P388/S or P388/VCR. Intraperitoneal administration of retinyl palmitate at 41.75 or 83.5 mg kg-1 was effective to potentiate the antileukaemic activity of VCR against P388/S bearing mice, and it also overcame vincristine-resistance in P388/VCR bearing mice.


Br. J. Cancer (1987), 56, 267–272

© The Macmillan Press Ltd., 1987

### Potentiation of vincristine by vitamin A against drug-resistant mouse leukaemia cells

I. Nogae, J. Kikuchi, T. Yamaguchi, M. Nakagawa, N. Shiraishi & M. Kuwano

*Department of Biochemistry, Oita Medical School, Hazama-cho, Oita 879-56, Japan.*

**Summary** Vitamin A has been shown to potentiate the cytotoxic action of anticancer agents like vincristine (VCR) against drug resistant mouse P388 leukaemia cells. *In vitro* tests showed enhancement by retinyl acetate of cytocidal activities of VCR against drug-sensitive leukaemia (P388/S) and VCR-resistant leukaemia (P388/VCR) cells in culture; retinyl acetate rather specifically potentiated VCR against cultured P388/VCR cells than P388/S cells. The cellular accumulation of radioactive VCR was significantly enhanced in cultured P388/VCR cells when retinyl acetate was present. The efflux of VCR from drug-resistant cells was blocked by retinyl acetate. The effect of the combination of vitamin A and VCR was also tested *in vivo* on the life-span of mice bearing P388/S or P388/VCR. Intraperitoneal administration of retinyl palmitate at 41.75 or 83.5 $mg\,kg^{-1}$ was effective to potentiate the antileukaemic activity of VCR against P388/S bearing mice, and it also overcame vincristine-resistance in P388/VCR bearing mice.

The development of resistance to anticancer agents is often a serious clinical problem in the treatment of cancer patients. The development of a methodology to overcome drug-resistance would be invaluable (Kuwano *et al.*, 1986). Drug resistance is circumvented in cultured mammalian tumour cells *in vitro* by various membrane active agents such as verapamil (Fojo *et al.*, 1985; Rogan *et al.*, 1984; Tsuruo *et al.*, 1981), lysosomotropic amines (Shiraishi *et al.*, 1986), isoprenoids (Nakagawa *et al.*, 1986) phenothiazine calmodulin inhibitors (Akiyama *et al.*, 1986) and a bisco-laurine alkaloid, cepharanthine (Shiraishi *et al.*, 1987). *In vivo* studies with animals carrying drug-resistant tumours have shown that verapamil (Tsuruo *et al.*, 1981) or isoprenoids (Yamaguchi *et al.*, 1986) are also effective. To apply such combination therapy clinically, the agents which overcome drug resistance should be nontoxic. As a plausible compound to combine, we used vitamin A which is a membrane active agent. Combination of vitamin A and anticancer agents has exhibited synergistic antitumour effects on various tumours *in vivo* (Akiyama *et al.*, 1981; Cohen & Carbonne, 1972; Nakagawa *et al.*, 1985; Tomita *et al.*, 1982). In this report, the effect of vitamin A on drug resistance in leukaemia cells is studied; some ability of vitamin A to overcome drug resistance is observed.

### Materials and methods

### *Cell line and cell culture*

P388/S, and its resistant subline P388/VCR, were obtained from Dr M. Inaba (Cancer Chemotherapy Cancer, Japanese Foundation for Cancer Research, Tokyo, Japan). This resistant subline was developed by *in vivo* treatment of drug-sensitive P388 leukaemia cells (P388/S) with VCR (Inaba *et al.*, 1979). Both sublines were passaged weekly by i.p. inoculation in BALB/c × DBA/2 (CDF1) mice (Shizuoka Agricultural Cooperative Association for Laboratory Animals, Hamamatsu, Japan) without drug injection. The relative resistance of each cell line to anticancer agents was confirmed for each assay because the phenotype of resistance is relatively unstable under *in vitro* conditions. Cells from P388/S or its resistant sublines were suspended in RPMI-medium (Grand Island Biochemical Co., Grand Island, NY, USA) supplemented with 10% foetal calf serum (FCS, Flow Laboratories, Inc., McLean, VA, USA) in the presence of 10 $\mu$M 2-hydroxyethyldisulfide (Aldrich Chemical Co., Inc., Milwaukee, WI, USA) and 100 $\mu g\,ml^{-1}$ kanamycin (Yamaguchi *et al.*, 1986).

### *Chemicals*

Radioisotopic compounds and chemicals were obtained from the following sources: [$^3$H] vincristine (VCR, specific activity, 4.8 $Ci\,mmol^{-1}$) from Amersham, UK; VCR and retinyl acetate (RA) from Sigma Chemical Co., St Louis, MO, USA; retinyl palmitate (RP), a gift from Hoffman-La Roche, Inc., Basel, Switzerland. All of the drugs except RP were freshly prepared by dissolving in dimethyl sulfoxide or absolute ethanol. Control experiments were done by adding the same amounts of dimethyl sulfoxide or ethanol. RP for animal experiments was dissolved in a detergent, HCO-60 (Nikko Chemical Co., Tokyo, Japan). All control experiments for *in vivo* trials were done by adding the same amounts of HCO-60.

### *In vivo antitumour activity*

P388/VCR was inoculated at $10^6$ cells per mouse i.p. into male $CDF_1$ mice. These mice were 5 to 6 weeks of age and weighed 18 to 22 g. Groups of 6 mice were housed in plastic cages and were given pelleted food and water *ad libitum* as described previously (Yamaguchi *et al.*, 1984, 1986). RP was injected i.p. daily from day 1 to 8. VCR dissolved in sterile pysiological saline was also administered simultaneously. The experiment was terminated on day 30. The therapeutic response was measured as the mean day of death of 6 mice group as described previously (Nakagawa *et al.*, 1985; Yamaguchi *et al.*, 1986).

### *Assay for growth curves to test circumvention of drugs resistance*

Cell survival was measured to test whether vitamin A could reverse drug resistance in P388 leukaemia cells *in vitro*. P388/S and P388/VCR were seeded in 24 multiwell plates at a cell density of $2 \times 10^5$ $cells\,ml^{-1}\,well^{-1}$. The cells were then exposed to various doses of VCR in the absence or presence of RA for 3 h at 37°C. Drug treatment with anticancer agents in the absence or presence of RA, 10% serum was either present or absent in the culture medium. Treatment for 3 h was found to show maximum effect, and no further enhancement of the circumventing effect was obtained after longer treatment than 3 h. The medium was then replaced with RPMI-1640 containing 10% FCS, and the cells were further incubated for 4 days at 37°C in the absence of drugs. Cell number was then measured in a model ZB1 Coulter Counter.

Correspondence: M. Kuwano
Received 27 November 1986; and in revised form, 14 April 1987.

*Assay for colony formation in soft agar to test circumvention of drug resistance*

Soft agar clonogenic assay was performed after Shoemaker *et al.* (1985). One ml of cell suspension of P388/S ($2 \times 10^4$ cells) and P388/VCR ($10^5$ cells) in RPMI medium containing 0.4% agar (Difco, Agar Noble), 10% FCS, 10 $\mu$M 2-hydroxy-ethyldisulfide and various doses of VCR or/and RA was added onto a 1 ml of base layer containing 0.5% agar, 10% foetal calf serum in RPMI in 35 mm plastic dishes. They were further incubated for 10 days at 37°C and colonies containing > 10 cells were scored.

*Drug accumulation*

The cells were seeded at $1 \times 10^6$ ml$^{-1}$ in 24 well of multi-well plates and treated with various doses of RA in RPMI-1640 without serum for 3 h at 37°C. Control plates were also prepared for each assay. The cells were incubated for 1 h with 1 ml of 26 nM [$^3$H] VCR, and then 1 ml of ice-cold PBS (g l$^{-1}$; NaCl, 8,0; $Na_2HPO_4 . 12H_2O$, 2.9; KCl, 0.2; $KH_2PO_4$, 0.2) was added to each well at 4°C. The cell pellets were harvested by centrifugation and washed three times with PBS, suspended in 0.9 ml of $H_2O$ and 10 ml of Scintisol EX-H (Wako Chemical Co., Osaka, Japan), and their radioactivity was then measured (Nakagawa *et al.*, 1986).

*Drug efflux assay*

Cells growing exponentially at $5 \times 10^5$ per well and control plates were incubated in the absence or presence of RA for 3 h at 37°C in RPMI-1640 without serum. Then P388/S in the presence or absence of RA and P388/VCR in the presence of RA were treated with 5.2 nM [$^3$H] VCR for 1 h at 37°C. For P388/VCR in the absence of RA, incubation was with 26 nM of [$^3$H] VCR to achieve the equivalent level of radioactive VCR accumulation. After 1 h incubation, the cells were washed once with ice-cold PBS at 4°C and efflux was followed over 120 min at 37°C in serum-free and radio-isotope-free medium with or without RA. At the time indicated, cells were harvested and their radioactivity was determined (Nakagawa *et al.*, 1986).

*Drug influx assay*

Cells growing exponentially at a density of $5 \times 10^5$ per well and control plates were incubated in glucose-free and serum-free HANKS balanced salt solution (HBSS) and exposed to various doses of RA for 3 h. The cells were further incubated with 1 mM 2,4-dinitrophenol for 10 min to inhibit drug efflux and followed by exposure to 26 nM [$^3$H] VCR for 1 min at 37°C (Nakagawa *et al.*, 1986; Shiraishi *et al.*, 1987). The cells were then harvested by centrifugation and the radioactivity associated with them determined.

### Results

*Effect of combination of vitamin A on VCR resistance of mouse leukaemia cells in culture*

P388/VCR is established by repeated administration of VCR to P388/S leukaemia bearing mice (Inaba & Johnson, 1978). To determine drug effects in the *in vitro* assay with P388/VCR or P388/S cells, we measured cell survival by constructing cell growth curves and clonogenic assay. We first treated P388/S or P388/VCR cells in the presence of VCR or/and RA for 3 h in medium with or without 10% serum, washed with drug-free fresh medium and followed incubation in 10% serum-supplemented medium in the absence of any drug. The cellular sensitivity to drugs of P388/VCR was compared with that of the parental P388/S by assaying the growth inhibition *in vitro* at 4th day after the treatment. We examined the effect of VCR alone or with RA on growth of P388/S and P388/VCR when treatment for 3 h with drugs was performed in the presence of serum. Differences in drug sensitivity to VCR was observed between P388/S and P388/VCR: P388/S cell growth was inhibited 50% by 50 ng VCR ml$^{-1}$, whereas growth of P388/VCR was inhibited by 50% at 2,000 ng VCR ml$^{-1}$ (Figure 1a). P388/VCR thus shows 20- to 40-fold greater resistance to VCR than the sensitive P388/S. Cellular sensitivity to RA was found to be similar between P388/S and P388/VCR: the surviving fraction of both P388/S and P388/VCR was decreased by only 10% of the initial fraction when 20 $\mu$g RA ml$^{-1}$ was present. Figure 1 also shows that RA partially overcomes VCR resistance in P388/VCR. The sensitivity of P388/VCR to VCR was enhanced 2- to 4-fold higher when 10 or 20 $\mu$g ml$^{-1}$ RA was present. By contrast, the cellular sensitivity of P388/S to VCR was not significantly enhanced by RA (Figure 1a). Some partial effect of RA on the circumvention of VCR resistance in mouse leukaemia cells was observed. Serum contains vitamin A binding proteins like retinol binding protein or other related proteins (Goodman, 1984), and serum might therefore interfere with the circumventing effect of RA against the leukaemia cells. We thus treated the cells with VCR or/and RA for 3 h under serum-free conditions, and then constructed the growth curves. As seen in Figure 1b, the circumventing effect of RA against P388/VCR was magnified. The $IC_{50}$ for P388/VCR was 2,000, 600 and 800 ng VCR ml$^{-1}$ in the presence of 0, 10 and 20 $\mu$g RA ml$^{-1}$, indicating that RA enhanced the cellular sensitivity of the resistant cells from 8- to 25-fold. The cellular sensitivity of P388/S to VCR was slightly (2- to 3-

**Figure 1** Effect of RA on resistance to VCR in P388/VCR cells. Exponentially growing P388/S (○, △, □) and P388/VCR (●, ▲, ■) cells were seeded either in 10% serum-supplemented medium (a) or in serum-free medium (b), and the cells were exposed to various doses of VCR in the absence (○, ●) or in the presence of 10 $\mu$g ml$^{-1}$ (△, ▲) and 20 $\mu$g ml$^{-1}$ (□, ■) of RA for 3 h. The cells were then followed by incubation for a further 4 days in a drug-free and fresh serum-supplemented medium. Each value is the average of duplicate dishes.

fold) enhanced in the presence of 10 and 20 μg RA ml$^{-1}$ (Figure 1b). Figure 2 shows an example of growth curves of P388/VCR in the absence of RA at 0, 10 and 20 μg ml$^{-1}$. Although RA alone at 10 or 20 μg ml$^{-1}$ had a slight effect on the growth of P388/VCR (Figure 2a), combination with VCR showed a dramatic reduction in the growth rate (Figure 2b).

We also tested the effect of RA on drug resistance in P388/VCR leukaemia cells by clonogenic assay in soft agar. Since colony forming ability of P388/VCR was found to be about 1/5 of that of P388/S, we plated a 5-fold greater number of P388/VCR cells than P388/S cells into each dish. As seen in Table I, the surviving fraction of P388/VCR synergistically decreased when combined with 10 μg ml$^{-1}$ RA. RA enhanced the cellular sensitivity of P388/VCR to VCR more than 4-fold when 10 or 40 ng ml$^{-1}$ VCR was present (Table I). RA alone at 10 μg ml$^{-1}$ only slightly affected the cell survival of P388/VCR as well as P388/S. RA also enhanced the cellular sensitivity to VCR of P388/S cell when 1 ng ml$^{-1}$ of VCR and RA were combined. Both assays for measuring cell survival by cell growth and colony formation showed a circumventing effect against VCR-resistant leukaemia cells by vitamin A.

**Figure 2** Effect of RA on cell growth of P388/VCR cells in the absence of the presence of VCR. Growth of P388/VCR cells was followed in serum-supplemented medium after exposure to 0 (○), 10 μg ml$^{-1}$ (△) and 20 μg ml$^{-1}$ (□) of RA in the absence (a) or presence (b) of 1 μg ml$^{-1}$ VCR for 3 h.

**Table I** Effect of RA and VCR on colony formation of P388/S and P388/VCR[a]

| | | *VCR (ng ml$^{-1}$)* | | | |
|---|---|---|---|---|---|
| *Cell lines* | *RA (10 μg ml$^{-1}$)* | *0* | *1* | *10* | *40* |
| P388/S | – | 100 | 24.8 ± 4.6 | 0 | 0 |
| P388/S | + | 100 | 10.2 ± 3.4 | 0 | 0 |
| P388/VCR | – | 100 | 87.5 ± 10.6 | 63.0 ± 8.0 | 31.3 ± 4.1 |
| P388/VCR | + | 100 | 50.0 ± 8.1 | 14.0 ± 4.3 | 0 |

[a]Relative survival fraction (%) was presented when colony number appearing in the absence or presence of RA without VCR was normalized as 100%. The colony number in the absence of RA was 2058 (P388/S) and 2352 (P388/VCR), and that in the presence of 10 μg ml$^{-1}$ RA was 1979 (P388/S) and 1568 (P388/VCR). Each value is average ± s.d. from triplicate dishes.

### *Effect of vitamin A on cellular accumulation of VCR in P338/S and P338/VCR cells*

To explore how vitamin A overcomes the drug resistance of P388/VCR, its effect on the cellular accumulation of anticancer agents as examined. Kinetics for VCR accumulation were observed for 4 h when P388/S and P388/VCR were incubated with [$^3$H] VCR at 37°C. The accumulation of [$^3$H] VCR reached saturation at 1 h in P388/VCR or P388/S cells. We compared dose-response effects of RA on the cellular accumulation of [$^3$H] VCR in P388/S and P388/VCR. Both cell lines were first incubated with various doses of RA, then [$^3$H] VCR was added to the medium for 1 h. The intracellular level of VCR in P388/VCR was approximately one-third of that in P388/S. Treatment with RA at 10 to 50 μg ml$^{-1}$ enhanced the accumulation of radioactive VCR in P388/VCR cells by 2- to 3-fold (Figure 3). Treatment with vitamin A at these doses reduced cell survival by < 10% of control. Figure 3 also showed enhancement of VCR accumulation by RA in the sensitive P388/S cells although the level of enhancement was only ~125%.

Enhanced outward transport (efflux) has been shown to be associated with drug resistance in mouse P388 leukaemia cells resistant to anticancer agents (Inaba & Johnson, 1978; Inaba *et al.*, 1979). We determined whether increased accumulation of anticancer agents in the resistant leukaemia cells by vitamin A is due to altered efflux and/or influx. To test the effect of vitamin A on drug efflux activity, the cells were exposed to [$^3$H] VCR for 60 min, followed by incubation with or without 50 μg RA ml$^{-1}$ in medium containing no isotope (Figure 4). More than 50% of cell associated radioactive VCR in P388/VCR cells was released into medium after 30 min of incubation in the absence of RA, and then there was a slight subsequent release of the radioactivity at 37°C. In contrast, in the parental cells, the release of VCR

**Figure 3** Effect of RA on drug accumulation in P388/S and P388/VCR. P388/S (□) and P388/VCR (▨) were seeded and treated with various doses of RA. P388/S and P388/VCR were then incubated with [$^3$H] VCR, and the cell-associated radioactivity was counted.

Figure 4 Effect of RA on drug efflux. P388/S (○, ●) and P388/VCR (△, ▲) were seeded and incubated in the presence or absence of 50 μg ml$^{-1}$ of RA. After the cells were further incubated with [$^{3}$H] VCR, they were exposed to assay medium in the absence (○, △) or presence (●, ▲) of 50 μg RA ml$^{-1}$. The values are mean ± s.d. from triplicate dishes.

Figure 5 Effect of RA on drug influx. P388/S (□) and P388/VCR (▨) pre-exposed to various doses of RA were further incubated with 1 mM 2,4-dinitrophenol, and then exposed to [$^{3}$H] VCR. The values are mean ± s.d. from triplicate dishes.

into medium proceeded much more slowly, and >50% of the initial activity still remained after 90 min incubation (Figure 4). Efflux of VCR from P388/VCR was significantly blocked by 50 μg RA ml$^{-1}$: more than 60% of the initial activity remained in RA-treated P388/VCR cells even after 120 min incubation (Figure 4). In the sensitive P388/S cells, treatment with RA did not significantly interfere with drug efflux.

The effect of RA on cellular uptake (influx) of VCR was also examined (Figure 5). Treatment of P388/VCR with 2,4-dinitrophenol increased accumulation of VCR only slightly if at all (c.f. Figures 3 and 5). As a function of dose of RA, accumulation of VCR into P388/VCR and P388/S increased about 2-fold (Figure 5).

*Effect of vitamin A on antitumour activity of VCR in P388/S- and P388/VCR-bearing mice*

We tested the *in vivo* effect of vitamin A to potentiate VCR against drug-resistant leukaemia bearing mice. Since RA is toxic and unstable *in vivo*, we used RP for *in vivo* tests. Our previous study showed that RP at ≥167 mg kg$^{-1}$ produced synergistic antitumour activity of various anticancer agents against P388/S bearing mice (Nakagawa *et al.*, 1985). We used various doses of RP ranging from 41.75–83.5 mg kg$^{-1}$. These doses of RP alone were found to be nontoxic in mice. We first tested the *in vivo* effect of RP and VCR on VCR-resistant P388/VCR bearing mice. VCR at 5, 10 and 30 μg kg$^{-1}$ administered daily for 8 days starting from day 1 increased the life-span of P388/S bearing mice significantly, up to 2-fold (Table II).

We then tested the effect of RP and VCR treatment on P388/VCR bearing mice. RP alone at 41.75–83.5 mg kg$^{-1}$ gave no therapeutic effect (Table II). To obtain similar therapeutic effects on P388/VCR bearing mice as on P388/S bearing mice by VCR alone, 3-fold to 6-fold higher doses of VCR were required. The combination of VCR with RP synergistically increased the life-span in all combination trials

**Table II** Effect of RP on antitumour activity of VCR in P388/S and P388/VCR bearing mice[a]

| *Drug and dosage* | *Survival time*[b] *(days)* | *T/C*[c] (%) | *T/V*[d] (%) |
|---|---|---|---|
| A. P388/S | | | |
| Control | 9.7 ± 0.8 | 100 | |
| RP, 83.5 mg kg$^{-1}$ | 10.7 ± 1.0 | 110 | |
| RP, 41.75 mg kg$^{-1}$ | 9.8 ± 0.4 | 93 | |
| VCR, 30 μg kg$^{-1}$ | 15.7 ± 0.8 | 162 | 100 |
| + RP, 83.5 mg kg$^{-1}$ | 20.8 ± 1.8** | 214 | 132 |
| + RP, 41.75 mg kg$^{-1}$ | 18.2 ± 2.8** | 188 | 116 |
| VCR, 10 μg kg$^{-1}$ | 13.8 ± 1.0 | 142 | 100 |
| + RP, 83.5 mg kg$^{-1}$ | 19.8 ± 1.3** | 204 | 144 |
| + RP, 41.75 mg kg$^{-1}$ | 15.8 ± 1.5** | 163 | 115 |
| VCR, 5 μg kg$^{-1}$ | 12.2 ± 0.8 | 126 | 100 |
| + RP, 83.5 mg kg$^{-1}$ | 15.8 ± 3.1** | 163 | 129 |
| + RP, 41.75 mg kg$^{-1}$ | 13.7 ± 0.8 | 141 | 112 |
| B. P388/VCR | | | |
| Control | 12.5 ± 0.7 | 100 | |
| + RP, 83.5 mg kg$^{-1}$ | 14.0 ± 2.7 | 112 | |
| + RP, 41.75 mg kg$^{-1}$ | 12.0 ± 1.3 | 96 | |
| VCR, 200 μg kg$^{-1}$ | 16.2 ± 1.7 | 130 | 100 |
| + RP, 83.5 mg kg$^{-1}$ | 19.3 ± 2.1** | 154 | 118 |
| + RP, 41.75 mg kg$^{-1}$ | 20.5 ± 2.7 | 164 | 126 |
| VCR, 100 μg kg$^{-1}$ | 15.3 ± 1.0 | 122 | 100 |
| + RP, 83.5 mg kg$^{-1}$ | 20.7 ± 2.3** | 166 | 136 |
| + RP, 41.75 mg kg$^{-1}$ | 17.8 ± 1.5** | 142 | 116 |
| VCR, 30 μg kg$^{-1}$ | 13.7 ± 1.4 | 110 | 100 |
| + RP, 83.5 mg kg$^{-1}$ | 16.7 ± 1.2** | 134 | 122 |
| + RP, 41.75 mg kg$^{-1}$ | 16.8 ± 1.8** | 134 | 122 |

[a]CDF$_1$ male mice were given i.p. implants of $10^6$ cells of P388/S or P388/VCR leukaemia on day 0 and drug was given i.p. daily from day 1 to 8. Each treated group comprised 6 mice and the controls, 20 mice. [b]**$P < 0.05$ by Student's *t* test as compared to VCR alone. [c]T/C (%) – increase in mean survival, treated/control × 100. [d]T/V (%) at each dose of VCR, the mean survival time of the treated group divided by the mean survival time of the group treated with VCR alone.

of two different doses of VCR with RP by up to 36% (Table II).

### Discussion

Our present study is the first to demonstrate that vitamin A can circumvent drug resistance in tumour cells. RP partially overcomes drug resistance in P388/VCR leukaemia bearing mice while RA overcomes drug resistance in cultured drug-resistant leukaemia cells. RP itself appears to be less active *in vitro* (unpublished data), but it is actively transformed into retinol-type vitamin A *in vivo* which is supposed to potentiate anticancer agents (Akiyama *et al.*, 1981). Clonogenic assays and growth curves apparently indicate a circumventing effect of vitamin A against VCR-resistance *in vitro*. During growth curve assays, addition of serum was found to weaken the circumventing effect of RA during the short exposure to RA and VCR. Higher doses of RA than $20\,\mu g\,ml^{-1}$ (see Figure 1) were required to observe effective circumvention in the presence of serum during the treatment (unpublished data). Lipid-depleted serum was also found to weaken the effect of RA (unpublished data), suggesting that retinyl binding protein or other related protein(s) might interfere with the RA-induced circumvention of VCR resistance in leukaemia cells. Further study is necessary to clarify which component in serum is involved in the effect.

The underlying mechanism for drug resistance in tumour cells has not been completely determined. Decreased intracellular accumulation of anticancer agents has been proposed to involve the acquisition of drug resistance in tumour cells, and the decreased cellular levels of anticancer agents is due to enhanced efflux of drug by drug-resistant tumour cells (Danø, 1978; Inaba & Johnson, 1978; Skovsgaard, 1978). Decreased drug permeability has been considered to be involved in drug resistance (Biedler & Riehm, 1970; Inaba *et al.*, 1979; Siegfried *et al.*, 1985). Although it has remained unclear how the transport system is deranged in drug-resistant tumour cells, recent study suggests increased binding of vinblastine and its analogs to the high molecular weight surface membrane P-glycoprotein specific for multidrug resistant tumour cells (Cornwell *et al.*, 1986; Sofa *et al.*, 1986). Verapamil, a potent agent in the circumvention of multidrug resistance, inhibits the binding of vinblastine to the membrane protein (Cornwell *et al.*, 1986). It would be interesting to determine whether RA interacts with the specific glycoprotein implicated in multidrug resistance.

The effect of vitamin A on plasma membranes was earlier shown to involve decreased stability of the membrane phospholipid (Lucy, 1970; Lucy & Dingle, 1964). Alteration of membrane lipids might secondarily change the membrane transport system. In this study, RA inhibited efflux of VCR from P388/VCR cells, resulting in enhanced accumulation of VCR. Since efflux is enhanced in drug-resistant tumour cells, any agent which inhibits drug efflux might overcome drug resistance (Kuwano *et al.*, 1986). Vitamin A like RA overcomes drug resistance possibly through inhibition of drug efflux from P388/VCR cells. However, the drug efflux from drug-sensitive P388/S cells is not enhanced. The stimulatory effect of vitamin A on VCR accumulation in P388/S cells appears to be caused by enhanced uptake activity of the anticancer agent rather than its inhibitory effect on drug efflux. RP-induced potentiation *in vivo* of VCR against P388/S leukaemia bearing mice might be partly due to its stimulatory effect on the drug uptake activity. On the other hand, vitamin A and its analogues are powerful immunological adjuvants (Dressler, 1980; Glaser & Lotan, 1979; Tannock *et al.*, 1972). This immunoadjuvant activity of vitamin A might also have some influence on the vitamin A effect *in vivo* against P388/S as well as P388/VCR leukaemia bearing mice.

Our recent relevant study has shown that synthetic isoprenoids with 9- to 10-isoprene chains potentiate various anticancer agents (Ikezaki *et al.*, 1984; Yamaguchi *et al.*, 1984) and some of them overcome drug resistance to anticancer agents *in vivo* as well as *in vitro* (Nakagawa *et al.*, 1986; Yamaguchi *et al.*, 1986). The combination of anticancer agents with these isoprenoids is thus expected to be useful not only in overcoming drug resistance but also in the enhancement of the antitumour activities of anticancer agents. Concerning vitamin A or other retinoids, the combination of retinol type vitamin A with anticancer agents causes synergistic antitumour effects against various tumour cell systems (Akiyama *et al.*, 1981; Cohen & Carbone, 1972; Nakagawa *et al.*, 1985; Tomita *et al.*, 1982). On the other hand, blood levels of vitamin A including retinol have been shown to be decreased in patients with various cancers as compared to control groups (Atukorala *et al.*, 1986; Ibrahim *et al.*, 1977). Cancer patients with higher levels of serum retinol respond more favourably to chemotherapy than those with lower levels of retinol (Soukop & Calman, 1978). These reports suggest a close correlation between plasma retinol levels and efficacy of cancer chemotherapy. Anticancer combination therapy with vitamin A might be expected to produce improved antitumour effects against drug-sensitive and drug-resistant populations of cancer cells. Clinical trials of the vitamin A therapy will be necessary to verify the above prediction.

We thank Dr M.M. Gottesman (NCI, USA) for critical reading of this manuscript. This work was supported by a grant-in-aid for Cancer Research from Ministry of Education, Science and Culture of Japan, and also by Cancer Research Foundation Fund (1987).

### References

AKIYAMA, S., MASUDA, A., TABUKI, T., KUWANO, M. & KOMIYAMA, S. (1981). Enhancement of the antitumor effect of 6-mercaptopurine by vitamin A. *Gann*, **72**, 742.

AKIYAMA, S., SHIRAISHI, N., KURATOMI, Y., NAKAGAWA, M. & KUWANO, M. (1986). Circumvention of multiple-drug resistance in human cancer cells by thioridazine, and chloropromazine. *J. Natl Cancer Inst.*, **76**, 839.

ATUKORALA, S., BASU, T.K., DICKRSON, W.Y., DONALSON, D. & SAKULA, A. (1979). Vitamin A, zinc and lung cancer. *Br. J. Cancer*, **40**, 927.

BIEDLER, J.L. & RIEHM, H. (1970). Cellular resistance to actinomycin D in Chinese hamster cells *in vitro*; cross-resistance, radioautographic, and cytogenic studies. *Cancer Res.*, **30**, 1174.

COHEN, M.H. & CARBONE, P.P. (1971). Enhancement of the antitumor effects of 1,3-bis(2-chloroethyl)-1-nitrosourea and cyclophosphamide by vitamin A. *J. Natl Cancer Inst.*, **48**, 921.

CORNWELL, M.M., PASTAN, I. & GOTTESMAN, M.M. (1986). Certain calcium channel blockers bind specifically to multidrug-resistant human KB carcinoma membrane vesicles and inhibit drug binding to P-glycoprotein. *J. Biol. Chem.*, **261**, 7921.

DANØ, K. (1978). Active outward transport of daunomycin in resistant Ehrlich ascites tumor cells. *Biochim. Biophys. Acta*, **323**, 446.

DRESSLER, D.W. (1968). Adjuvanticity of vitamin A. *Nature*, **217**, 527.

FOJO, A., AKIYAMA, S., GOTTESMAN, M.M. & PASTAN, I. (1985). Reduced drug accumulation in multiply drug-resistant human KB carcinoma cell lines. *Cancer Res.*, **45**, 3002.

GLASER, M. & LOTAN, R. (1979). Augmentation of specific tumor immunity against a syngenic SV-40-induced sarcoma in mice by retinoic acid. *Cell Immunol.*, **45**, 175.

GOODMAN, D.S. (1984). Plasma retinol binding protein. In *The Retinoids*, Sporn, M.B., Roberts, A.B. & Goodman, D.S. (eds) p. 41. Academic Press: New York.

IBRAHIM, K., JAFAREY, N.A. & ZUVERI, S.J. (1977). Plasma vitamin A and carotene in squamous cell carcinomas of oral cavity and oro-pharynx. *Clin. Oncol.*, **3**, 203.

IKEZAKI, K., YAMAGUCHI, T., MIYAZAKI, C. & 8 others (1984). Potentiation of anticancer agents by new synthetic isoprenoids. I. Inhibition of the growth of cultured mammalian cells. *J. Natl Cancer Inst.*, **73,** 895.

INABA, M. & JOHNSON, R.K. (1978). Uptake and retention of Adriamycin and Daunorubicin by sensitive and anthracycline-resistant sublines of P388 leukemia. *Biochem. Pharmacol.*, **27,** 2123.

INABA, M., KOBAYASHI, H., SAKURAI, Y. & JOHNSON, R.K. (1979). Active efflux of daunomycin and Adriamycin in sensitive and resistant sublines of P388 leukemia, *Cancer Res.*, **39,** 2200.

KUWANO, M., NAKAGAWA, M., SHIRAISHI, N. & 3 others (1986). Techniques to reverse or circumvent drug-resistance *in vitro*. In *Cancer Drug Resistance,* Hall, T. (ed) p, 163. Alan R. Liss, Inc.: New York.

LUCY, J.A. (1970). The fusion of biological membranes. *Nature*, **227,** 815.

LUCY, J.A. & DINGLE, J. (1964). FAT-soluble vitamins and biological membranes. *Nature*, **204,** 156.

NAKAGAWA, M., AKIYAMA, S., YAMAGUCHI, T. & 3 others (1986). Reversal of multidrug resistance by synthetic isoprenoids in human cancer cell line. *Cancer Res.*, **46,** 4453.

NAKAGAWA, M., YAMAGUCHI, T., UEDA, H. & 5 others (1985). Potentiation by vitamin A of the action of anticancer agents against murine tumors. *Jpn. J. Cancer Res.*, (Gann), **76,** 887.

ROGAN, A.M., HAMILTON, T.C., YOUNG, R.C., KLECKER, R.W. & OZOLS, R.F. (1984). Reversal of Adriamycin resistance by verapamil in human ovarian cancer. *Science*, **224,** 994.

SHIRAISHI, N., AKIYAMA, S., KOBAYASHI, M. & KUWANO, M. (1986). Lysosomotropic agents reverse multiple drug resistance in human cancer cells. *Cancer Lett.*, **30,** 251.

SHIRAISHI, N., AKIYAMA, S., NAKAGAWA, M., KOBAYASHI, M. & KUWANO, M. (1987). Effect of bisbenzylisoquinoline (biscolaurine) alkaloids on multidrug resistance in KB human cancer cells. *Cancer Res.*, **47,** 2413.

SHOEMAKER, R.H., WOLPERT-DEFILIPPES, M.K., KERN, D.H. & 8 others (1985). Application of a human tumor colony-forming assay to new drug screening. *Cancer Res.*, **45,** 2145.

SIEGFRIED, J.M. & BURKE, T.R. (1985). Cellular transport of anthracyclines by passive diffusion. Implication for drug resistance. *Biochem. Pharmacol.*, **34,** 593.

SKOVSGAARD, T. (1978). Mechanism of resistance to daunorubicin in Ehrlich ascites tumor cells. *Cancer Res.*, **38,** 1785.

SOFA, A.R., GLOVER, C.J., MAYERS, M.B., BIEDLER, J.L. & FELSTED, R.L. (1986). Vinblastine photoaffinity labeling of a high molecular weight surface membrane glycoprotein specific for multidrug-resistant cells. *J. Biol. Chem.*, **261,** 6137.

SOUKOP, M. & CALMAN, K.C. (1978). Vitamin A status and chemotherapeutic responses in cancer patients. In *Current Chemotherapy. Proc. 10th Int. Congr. Chemother.*, Siegenthaler, W. & Luthy, R. (eds) p. 1296. Zurich.

TANNOCK, I.F., SUIT, H.D. & MARSHALL, N. (1972). Vitamin A and radiation response of experimental tumors: An immunemediated effect. *J. Natl Cancer Inst.*, **48,** 731.

TOMITA, Y., HIMENO, K., NOMOTO, K., ENDO, H. & HIROHATA, T. (1982). Combined treatments with vitamin A and 5-fluorouracil and the growth of allotransplantable and syngeneic tumors in mice. *J. Natl Cancer Inst.*, **68,** 823.

TSURUO, T., IIDA, H., TSUKAGOSHI, S. & SAKURAI, Y. (1981). Overcoming of vincristine resistance in P388 leukemia *in vivo* and *in vitro* through enhanced cytotoxity of vincristine and vinblastine by verapamil. *Cancer Res.*, **41,** 1967.

YAMAGUCHI, T., IKEZAKI, K., KISHIYE, T. & 7 others (1984). Potentiation of anticancer agents by new synthetic isoprenoids. II. Inhibition of the growth of transplantable murine tumors. *J. Natl Cancer Inst.*, **73,** 903.

YAMAGUCHI, T., NAKAGAWA, M., SHIRAISHI, N. & 5 others (1986). Overcoming drug resistance in cancer cells with synthetic isoprenoids. *J. Natl Cancer Inst.*, **76,** 947.